# Investigating different dimensions of infertile women’s quality of life: a descriptive cross-sectional study

**DOI:** 10.1186/s12889-022-14924-w

**Published:** 2022-12-27

**Authors:** Zahra Kiani, Masoumeh Simbar, Sepideh Hajian, Farid Zayeri, Farzaneh RashidiFakari, Fatemeh Jalali Chimeh

**Affiliations:** 1 Midwifery and Reproductive Health Research Center, Shahid Beheshti University of Medical Sciences, Tehran, Iran; 2 Midwifery and Reproductive Health Research Center, Department of Midwifery, School of Nursing and Midwifery, Shahid Beheshti University of Medical Sciences, Tehran, Iran; 3 Department of Midwifery, School of Nursing and Midwifery, Shahid Beheshti University of Medical Sciences, Tehran, Iran; 4 Proteomics Research Center and Department of Biostatistics, Faculty of Allied Medical Sciences, Shahid Beheshti University of Medical Sciences, Tehran, Iran; 5 Department of Midwifery, School of Medicine, North Khorasan University of Medical Sciences, Bojnurd, Iran

**Keywords:** Quality of life, Women, Infertility, Iran

## Abstract

**Background:**

Infertility is a major challenge in the life of women which affects their quality of life. Infertile women's quality of life is a relatively new field of research that has recently been considered by health researchers. However, there has been no standard tool for measuring different aspects of infertile women's quality of life with female factors, and general and specific tools of infertile couples have been used to assess their quality of life. This study, thus, aimed to analyze different aspects of the quality of life of infertile women.

**Methods:**

This descriptive cross-sectional study was conducted on 320 infertile women referred to a teaching hospital affiliated with Mazandaran University of Medical Sciences and private infertility treatment centers in Sari, Iran. Demographic and fertility characteristics and the quality of life questionnaire for infertile women questionnaire (a 25-item tool was designed which measured 7 factors of psychological effects, sexual life with infertility family and social effects, infertility-related concerns, physical effects, adaptive approaches and factors preventing infertility adaptation), were recruited for data gathering. Data were analyzed using SPSS version 22. Descriptive statistics (percentage, mean, standard deviation), correlation coefficient, independent sample t-test, and multiple linear regression were used. *P*-values less than 0.05 were considered statistically significant.

**Results:**

The total mean score of infertile women's quality of life was 65.68 ± 8.91%. Findings were indicative of infertile women's quality of life in the dimensions of adaptive approach (70.48 ± 15.02%), psychological (67.88 ± 12.06%), family and social (64.63 ± 10.76%), physical, 63.42 ± 11.36%), inhibitory factors/ factors preventing adaptation (60.98 ± 8.24%), related concerns (51.52 ± 10.21%) and sexual life (40.12 ± 14.28%). According to the final multiple linear regression model, women's education (B = 2.57, *p* < 0.001), spouse's education (B = 1.56, *p* = 0.046), economic status (B = 1.64, *p* < 0.001), age of women (B = -0.62, *p* < 0.001), age of spouse (B = -0.65, *p* < 0.001), duration of infertility (B = -0.36, *p* = 0.024) and duration of marriage (B = -0.39, *p* = 0.022) were the final predictors of the quality of life score in infertile women of the study.

**Conclusion:**

Given that infertility causes extensive changes in individuals, families, and social dimensions of infertile women, it can affect their quality of life. We can take steps to improve the health of infertile women by promoting various dimensions of their quality of life.

**Supplementary Information:**

The online version contains supplementary material available at 10.1186/s12889-022-14924-w.

## Background

Infertility refers to the lack of pregnancy after one year of a sexual relationship in absence of any contraceptive method [1]. The prevalence of infertility has increased compared to previous decades [2] and, according to the World Health Organization (WHO), there are about 60 to 80 million infertile couples worldwide and the prevalence of it varies in different parts of the world [3]. The results of a recent study in Iran have shown that the prevalence of infertility in Iran (20.2%) is higher than the global average and one-fifth of Iranian couples are infertile. As such, infertility is supposed to be a national problem [4].

Infertility causes women to experience changes in various aspects of their lives and leads to emotional, psychological, and social disorders. Moreover, there is a conflict between worries and coping strategies in infertile women that can affect their quality of life [5]. Infertile women experience different personal, family, and social problems. A review of studies in many parts of the world showed that women carry the main burden of infertility and have to tolerate a great deal of anxiety as they are blamed for their inability to childbearing [6]. Infertile women face the high costs of infertility treatments, frequent doctor visits, and scheduled sexual intercourse that affect their quality of life [5]. Given the fact that pregnancy is one of the most important goals of women, infertility can instill a sense of lack and deficiency in infertile women [7].

Furthermore, infertility can decrease sexual attractiveness and sexual desire in women. Change in one's sexual desires is a significant issue that can affect their quality of life [8]. However, few studies have shown that the quality of marital relationships in infertile couples is higher than in fertile ones, and infertility causes couples to get closer to each other and talk more about their worries in the future [9, 10].

At a social level, many infertile women suffer from social isolation and despair. In developing societies, a woman is considered complete only when she becomes a mother. Causing inequality between men and women, this issue leads to gender-related suffering among women [11]. Infertility, as a general stigma, brings about devastating consequences such as feelings of insignificance and worthlessness for infertile women, which may in turn affect their quality of life [12].

There are various definitions for quality of life. Researchers usually define the quality of life by focusing on one of the three areas of well-being and peace in life, economic and social power and physical symptoms, illness, and disability [13]. The WHO defines quality of life as people's perceptions of their position in life in terms of the cultural systems and values ​​in which they live, and how these perceptions are related to their goals, expectations, standards, and concerns. This concept is a very broad one which includes physical and mental health, autonomy level, social relationships, personal beliefs, and the individual's relationship with the environment [14]. Infertile women's quality of life is an important issue that has recently been considered by health researchers. Moreover, women play a pivotal role in different stages of individuals' life and hence, their quality of life can significantly affect the health of the individual and society [15].

Focusing on clinical aspects of infertile women's quality of life, a cross-sectional study surveyed 106 infertile women who were referred to infertility centers in Brazil in 2020. The 36-Item Short Form Survey (SF-36) Quality of Life Questionnaire was used in this study to assess the quality of life of infertile women. According to the results, the scores obtained by infertile women were low and the increased duration of infertility was shown to have an adverse effect on their quality of life [16]. Massarotti et al. conducted a study in 2019 to evaluate the effect of infertility treatments on quality of life as well as on the level of anxiety and depression in infertile women in three groups based on the type of infertility factor (male, female, and both factors). They used the Hospital Anxiety and Depression Scale (HADS) and the Fertility Quality of Life questionnaire (FertiQoL). The findings showed that infertile women with female factors had lower scores of quality of life compared to the other two groups. Moreover, their scores for anxiety and depression were high [17]. Similar results were obtained in the study of Jahromi et al. where the FertiQoL questionnaire was used [18]. In a systematic review study, it was noted that although infertility had a negative effect on couples' mental health and sexual relationships, it did not affect the quality of life of infertile couples [19].

In studies conducted around the world, data collection tools for assessing the quality of life of infertile women are general tools such as World Health Organization Quality of Life (WHOQOL) [14] and SF-36 [20] or specific questionnaires for infertile couples such as Quality of Life in Infertile Couples Questionnaire (QOLICQ) by Yaghmaei et al. (2009) [21] and FertiQoL by Boivin et al. (2011) [22]. On the one hand, general tools are not suitable for measuring changes in illness or health problems and, on the other, specific tools ignore the type of infertility factor which, according to the literature review, can affect the quality of life. Given the significance of accurate measurement of the quality of life dimensions in infertile women with the female factor, we decided to use a standard tool designed for assessing the quality of life of infertile women with female factor and examine the different dimensions of the quality of life of infertile women with the female factor [23]. The quality of life questionnaire for infertile women (QOL-QIW) measures the quality of life and its aspects by the seven dimensions of the questionnaire, including 1) Psychologic; 2) Sexual life; 3) Family and social; 4) Infertility-related concerns; 5) Physical; 6) Adaptive approaches; and 7) Preventive factors of adaptation [23]. This tool helps to show the needs in different dimensions of these women's quality of life and also to plan future needs-based interventions to improve the women's quality of life. Since the quality of life of infertile women has not been assessed with a valid tool yet, this study aimed to assess the quality of life of women with female infertility factors by using QOL-QIW in Iran.

## Methods

### Type of study

This descriptive cross-sectional study was conducted from June to December 2020.

### Participants and the research setting

The participants included infertile women with female infertility who have referred to a teaching hospital affiliated with Mazandaran University of Medical Sciences and private infertility centers in Sari, Iran. The hospital is the largest medical training center of Northern University of Medical Sciences of Iran and the referral center of the province and neighboring provinces in the fields of neurosurgery, gynecology, obstetrics, IVF, vascular, thoracic, multiple trauma, orthopedics, cancer, and super-specialized center for all internal trends, ICU and NICU.

### Inclusion criteria for the research subjects were as follows

One year of infertility with female factor diagnosed by a specialist, women whose infertility was not due to male factor, formal and permanent marriage, living currently with their spouses, no adoption, no severe mental illness (including major depression, schizophrenia, and mania), interested in participating in the study, no experience of mourning in the last 6 months and no use of psychiatric medication at the time of the study.

### Exclusion criteria

Unwillingness to participate in the study.

### Sample size

Using the following formula and a pilot study on 40 infertile women with a mean quality of life score of 79.78%, standard deviation of 14.5, and accuracy of 0.2, the sample size was calculated to be 320.

$$n=\frac{Z^{2}_{a/2}\cdot S^2}{d^2}$$

### Sampling method

The convenience sampling method was used in this study.

### Data collection tool

The questionnaire of demographic characteristics and a valid and reliable quality of life questionnaire for infertile women (QOL-QIW), designed by Kiani et al. [23], were used in this study.

### Demographic and fertility characteristics questionnaire

The validity of this 12-item questionnaire was assessed by experts (reproductive health specialist, psychologist, psychiatrist, midwife, gynecologist, and quality of life specialist). The questions of this questionnaire consist of information about age, education, occupation, economic status, place of residence, duration of the marriage, duration of infertility, type of infertility, and infertility treatment.

### QOL-QIW

Infertile women's quality of life tool of Kiani et al. [23] was designed in a sequential exploratory study. In the first stage of this questionnaire, a qualitative study with a content analysis approach was used to explain the concept and dimensions of infertile women's quality of life. Then, in the second stage, a quantitative study with an inductive-deductive approach was designed and its psychometric properties were evaluated based on the codes extracted from the qualitative stage, expert opinion and literature review, quality of life questionnaire for infertile women by using the basic steps of Waltz et al. tool, which includes the selection of a conceptual model for identifying the components of the measurement process, explanation of the objectives for the measurement, creation of a roadmap or blueprint, as well as the development of the tool including the use of the mentioned steps, along with the regulation of the items and scoring rules. Finally, a 25-item tool was designed which measured 7 factors of psychological effects (4 items), sexual life with infertility (3 items), family and social effects (3 items), infertility-related concerns (5 items), physical effects (3 items), adaptive approaches (4 items) and factors preventing infertility adaptation (3 items), where the means scale- content validity index (S-CVI) and Item- content validity index (I-CVI) were 0.94 and 0.92 respectively, and the reliability of the tool was confirmed with the Cronbach's alpha of 0.87 and intra-cluster correlation of 0.97. The criteria for interpretability and ease of use were also acceptable. The options of the quality of life questionnaire for infertile women are based on a 5-point Likert scale. The points of the Likert scale include never (5), rarely (4), sometimes (3), most of the time (2), and always (1), and questions 19, 20, 21, and 22 are scored inversely. Each person's scores ranged from 0 to 100. 

The correlation coefficient between a generic instrument with the SF-36 questionnaire and a specific instrument the QOL-QIW was 0.61; considering that there was a correlation coefficient of 0.5 to 0.7 between the quality of life questionnaire for infertile women and the SF-36 questionnaire the concurrent validity was good.

### Data collection method

Given the prevalence of the Covid-19 pandemic and the high risks of virus transmission during the process of completing the questionnaires, electronic sampling was used through the Google platform. Going to a teaching hospital affiliated with Mazandaran University of Medical Sciences and private infertility treatment centers in Sari, the researcher identified the infertile women who were eligible for the study. After explaining the research objectives, obtaining written electronic informed consent, and getting their mobile number if they were willing, the electronic questionnaire was sent to them via WhatsApp application and completed by them in infertility centers or whenever they wanted. In cases where the subjects would not like to give their phone number, the researcher's phone, which was specifically used for this purpose, was provided to them considering health protocols.

### Data analysis method

The data were entered into the Statistical Package for the Social Sciences (SPSS) software version 22. Descriptive statistics were used for presenting and describing the information in tables and the calculation of percentage, mean and standard deviation. Inferential statistics were also used for analysis and the relationships. First, the distribution of quantitative variables was investigated using the Kolmogorov–Smirnov test, and parametric statistical tests were used in the case of the normal distribution; otherwise, nonparametric tests were used. The correlation coefficient, independent sample t-test, and multiple linear regression were used for data analysis. *P*-values less than 0.05 were considered statistically significant.

### Ethics approval

This study was approved by the ethics code of IR.SBMU.PHARMACY.REC.1400.002 in Shahid Beheshti University of Medical Sciences. All methods were carried out in accordance with relevant guidelines and regulations. Participation in the study was fully voluntary and contingent on consent and all methods were carried out under relevant guidelines and regulations. To maintain the confidentiality of participants’ data, the questionnaires were completed anonymously with no identification number. At the beginning of each questionnaire, the consent option to participate in the research was placed and all participants provided informed consent to include in the study.

## Results

The data of 320 infertile women showed that the mean age of them, the mean duration of the marriage, and the mean duration of infertility were 31.79 ± 5.58, 9.08 ± 3.61, and 4.21 ± 6.21 years respectively. Other demographic characteristics are shown in Table 1.

**Table 1:** Demographic characteristics of infertile women (*n* = 320

Variable	Class	No	%
Education	Illiterate	10	3.2
	Primary school	35	10.9
	Secondary school	38	11.9
	High school	83	25.9
	Bachelor's degree	112	35.0
	Master's degree	31	9.7
	PhD	11	3.4
Spouse education	Primary school	3	0.9
	Secondary school	23	7.1
	High school	77	24.1
	Bachelor's degree	148	46.3
	Master's degree	53	16.6
	PhD	16	5.0
Job	Homemaker	233	72.8
	Employed	87	27.2
Spouse job	Employee	111	34.9
	Manual worker	22	6.9
	Farmer	35	10.9
	Self-employed	140	43.8
	University student	2	0.7
	Others	10	2.8
Economic status	Weak	81	25.4
	Average	146	45.6
	Good	66	20.6
	Very good	27	8.4
Place of residence	Village	71	22.2
	City	271	77.8
History of family infertility	Yes	46	14.4
	No	274	85.6
Type of infertility	Primary	249	77.8
	Secondary	71	22.2
Type of treatment	Pharmacological	120	37.5
	IUI	86	26.8
	IVF	94	29.4
	Donated oocyte	20	6.3
Total		320	100

The mean score of infertile women's quality of life was 65.68 ± 8.91%. The highest mean score was in the dimension of adaptive approaches and the lowest one belonged to the dimension of sexual life with infertility (Table 2).

**Table 2:** Mean and standard deviation of infertile women's quality of life and its dimensions (*N* = 320)

Dimensions	Mean based on 0–100	SD
Psychological effects	67.88	12.06
Sexual life with fertility	40.12	14.28
Family and social effects	64.63	10.76
Infertility-related concerns	51.52	10.21
Physical effects	63.42	11.36
Adaptive approaches	70.48	15.02
Factors preventing adaptation	60.98	8.24
Total	65.68	8.91

As the results of the t-test shown in Table 3, there was a statistically significant difference in the quality of life scores based on the type of infertility in infertile women (*P* < 0.001), and women with primary infertility had a lower quality of life mean score. There were no significant differences in the quality of life of the women with a different family history of infertility, women’s jobs, and place of residence between groups (*P* > 0.05).

**Table 3:** The distribution of infertile women's quality of life scores based on the type of infertility (*N* = 320)

Type of infertility	Mean	SD
Primary	65.26	8.75
Secondary	67.17	9.38
Total	65.68	8.91
Type of the test	t-test	
Results	T = 3.08, *P* < 0.001	

Infertile women's quality of life had the highest positive and significant correlation with women's education (*P* < 0.001, *r* = 0.75). It had also a negative and significant correlation with the age of the women and their spouses, duration of the marriage, and duration of infertility. The highest inverse relationships were with the duration of marriage (*r* = -0.65) and the duration of infertility (*r* = -0.59) respectively (*P* < 0.001) (Table 4).

**Table 4:** The correlation between the infertile women's quality of life and their demographic characteristics (*N* = 320)

Variable	Correlation coefficient	Correlation with the infertile women's quality of life	P
Age of women	Pearson	-0.21	0.032
Age of spouses	Pearson	-0.41	0.021
Duration of marriage	Pearson	-0.65	*P* < 0.001
Duration of infertility	Pearson	-0.59	*P* < 0.001
Education of women	Spearman	0.75	*P* < 0.001
Education of spouses	Spearman	0.61	*P* < 0.001
Economic status	Spearman	0.50	*P* < 0.001

Multiple linear regression was used to determine the prediction of variables. First, the variance inflation index (VIF) was used to examine multicollinearity and the results showed that there was no overlap between the variables (VIF < 10). As the results showed, 65% of the variance of the quality of life total score in infertile women was explained by these variables.

According to the final multiple linear regression model, women's education (B = 2.57, < 0.001), spouse's education (B = 1.56, *p* = 0.046), economic status (B = 1.64, *p* < 0.001), age of women (B = -0.62, *p* < 0.001), age of spouse (B = -0.65, *p* < 0.001), duration of infertility (B = -0.36, *p* = 0.024) and duration of marriage (B = -0.39, *p* = 0.022) were the final predictors of the quality of life score in infertile women of the study (Table 5).

**Table 5:** Predictors of the infertile women's quality of life score (*N* = 320)

Variable	Unstandardized regression coefficient B	Standard error	(*p*-value)	The 95% confidence interval for coefficient B	
				Minimum	Maximum
Education level	2.57	0.61	< 0.001	1.374	3.766
Spouse education	1.56	0.78	0.046	0.031	3.089
Economic status	1.64	0.23	< 0.001	1.189	2.091
Age of women	-0.62	0.10	< 0.001	-0.816	-0.424
Age of spouse	-0.65	0.12	< 0.001	-0.885	-0.415
Duration of infertility	-0.36	0.16	0.024	-0.674	-0.046
Duration of marriage	-0.39	0.17	0.022	-0.723	-0.057

## Discussion

To the current knowledge of researchers, this study for the first time examined different dimensions of the quality of life of infertile women with female factors by using a specific tool. Using this tool, seven dimensions of psychological dimension, sexual life with infertility, family and social effects, infertility-related concerns, physical effects, adaptive approaches, and factors preventing adaptation were examined. According to the results, the quality of life scores of women was 65.68 ± 8.91percent and the highest and lowest scores were related to the adaptive approach and sexual dimension, respectively. Most studies have used questionnaires on the quality of life of infertile couples by Yaghmaei et al. (2009) and the quality of life of infertile couples by Boivin et al. (2011). As the findings of these studies have indicated, the quality of life of infertile women is lower than men, and disorders such as anxiety, social dysfunction, and depression strongly affect the quality of life of women [24–27]. Moreover, a systematic study reviewed the studies that used the WHOQOL and SF-36 questionnaires. According to the results of this review, the quality of life of infertile couples was reported to be low in two studies that had used the SF-36 questionnaire, while it was high in a study that had used the WHOQOL questionnaires. As the couples were more educated in this study, they felt that infertility had brought them closer and they could solve this problem together [19].

The mean score of infertile women was 67.88 ± 12.06 percent in the psychological dimension. Infertility often causes a lot of stress in women that may affect their quality of life [28]. In the study conducted by Moghadam et al. where they used the SF-36 questionnaire, the mean score of psychological dimension was 61.69 ± 17.78 percent [29]. The results of the study by Bornstein et al. (2020) showed that infertile women are more affected by psychological aspects of their lives than men and are more depressed and anxious as well [30]. Although these results have not been obtained in some studies [19, 31], most studies indicated that the mental vulnerability of infertile women is greater than men. In a meta-analysis study conducted by Kiani et al. (2020), the overall prevalence of anxiety in infertile women was reported to be 36.17%, which was 54.24% in middle- and low-income countries [32]. Another meta-analysis study revealed in 2021 that the overall prevalence of depression in infertile women was 39.78%, while it was 44.32% in low- and middle-income countries [33]. Having children and parenting are traditionally considered to be the most prominent features of the female gender role and that is why infertility is considered in traditional communities to be a feminine deficit. Additionally, infertility-related sufferings in women are more severe and deeper than in men. As such, women are supposed to be more vulnerable, and the social structures that cause this suffering and the resulting consequences that affect the quality of life of these women are different from infertile men. Therefore, women face different emotional problems which differentiate their quality of life from that of men [23]. Using this questionnaire, the current study examined this dimension specifically in women with a female factor.

Based on the results of our study, the women obtained the lowest score in the dimension of the quality of sexual life. Sexual behaviors of infertile women have different physiological, cultural, social, and family dimensions and are influenced by the process of diagnosis and level of infertility treatment, prevalent beliefs about infertility, sexual response cycle, and socio-cultural context [34]. Moreover, the nature of the sexual behaviors of infertile people may change and they do these behaviors only for obedience and childbearing not enjoying their sexual life [6]. Most studies have shown that infertility reduces the number of sexual intercourse [35, 36] and sexual desire and satisfaction [37, 38], leading to sexual dysfunction in couples [34, 39, 40]. There is no sexual attraction for infertile women and their sexual desire is reduced. As an important issue, sexual desire can affect one's quality of life [8]. However, it has been shown in many studies that infertile couples have a better marital relationship than fertile couples, and infertile couples are closer to each other as they try to solve their problems together and give comfort to each other [9, 10]. In their study, Kohan et al. investigated the sexual life of infertile people. They found that infertile women felt a kind of reduction of their feminine characteristics and deficit during infertility that had a negative effect on their sexual life [41]. According to many studies, as women are aware of their childbearing role, their inability in becoming mothers challenges their feminine characteristics, leading to some dysfunctions in their sexual relations [42].

The infertile women obtained a mean score of 64.63 ± 10.76 percent in the dimension of quality of family and social life. This dimension has also been referred to in other studies where infertile women obtained average scores. The issue of fertility is highly significant in most cultures and the desire for having a child is a human stimulus to continue living, and any lack of pregnancy can lead to adverse effects on family and social relationships [43]. Almin et al. have shown that infertility affects family relationships and, affected by the detrimental effects of infertility, many people try to stay away from their families [44]. As shown by some studies, women are more likely than men to bear the burden of infertility and are exposed to negative social stigmas which may affect their relationship with other family and society members [37, 45]. To hide the problem, most infertile women often try to stay away from family and friends [46]. It has also been argued in some studies that the concept of infertility and the social relations of infertile people change over time. Additionally, the hopes of infertility treatment have caused the social relations of many infertile people to not change much after infertility [5].

Infertility-related concerns obtained the lowest score after the sexual dimension, and the mean score of women was 51.52 ± 10.21 percent in this dimension. The questions of this dimension were concerned with the effect of age on infertility, repetition of treatment, and harms of assisted reproductive techniques on the fetus, which are not available in other questionnaires. Given the nature of the problem and the impact on quality of life, these items indicate that this tool is specifically designed for the quality of life of infertile women with a female factor and has specific questions for addressing their concerns. Becoming a mother and raising a child is a turning point in women's lives and lack of childbearing will cause them anxiety [47]. In most low- and middle-income countries, women are not employed and infertility treatment costs impose some burden on their husbands, leading to more anxiety in them [32]. Furthermore, infertility treatment requires frequent visits to the doctors and the use of various medications that, imposing great economic burdens, can affect the health of individuals and their quality of life [48, 49]. According to some studies, infertility can lead to divorce, which has occupied the mind of couples since the beginning of infertility and has increased their panic in this regard [50]. The low chance of remarriage for women and the condemnation of single life in traditional societies put additional pressure on women compared to men, causing more worry in them and affecting their quality of life [51). Moreover, as they do not have a child to take care of them in old age or during an illness, these women may be afraid of loneliness in the future [51]. Fear of loneliness in the future [51] and fear of divorce [52] are important factors that increase the incidence of depression in women and have negative effects on the infertility treatment response [53].

Physical dimension was another aspect of this questionnaire whose mean score was 63.42 ± 11.36 percent. In studies that used general and specific quality of life questionnaires, similar to our study, the score of the physical dimension of infertile women was lower than the overall total score [16, 54]. In fact, with its complex treatments and various stresses, infertility has been found characteristic of chronic physical diseases that cause physical fatigue in individuals [55]. Moreover, the medicines used in the treatment of infertility have often many gastrointestinal, neurological, cardiovascular, dermal, skeletal, and muscular side effects [56]. Sabarre et al. also have referred to weakness and lethargy of infertile people during the treatment process [57]. Similarly, Bakhtiyar et al. (2019) indicated that because of receiving infertility services, infertile women had frequent visits to infertility centers that could cause physical fatigue in them [58]. All of these factors can affect the quality of life of infertile women negatively.

The infertile women in our study obtained the highest score in the dimension of adaptive approach which was almost 5 points higher than the total mean score of quality of life. In this dimension, women received the highest percentage of score in the item related to trusting in God. Given the Iranian culture, religious beliefs play an important role in dealing with problems of life, based on which the high scores of women in this dimension can be partly justified. Although communication with God is one of the spiritual issues that plays a significant role in relieving people and helping them cope with infertility, its role is under discussion in different societies [59]. In some societies, childbearing is even considered to be a means of achieving piety [48]. Receiving social support from others and medical staff is one of the strategies that may help infertile women to deal with infertility [60]. Based on studies, infertile people who have received social support have a better quality of life and use better-coping strategies [61]. On the other hand, infertility is considered in some studies to be a social disgrace or stigma. The more the burden of this stigma in society, the more infertility is considered to be a threat to self-esteem. In such a condition, infertile women have less social support and their coping ability will be gradually reduced. Generally speaking, these problems are more prominent in women than men that may affect their quality of life [62].

As studies have shown, adaptive approaches have been among the ways of dealing with infertility. Although infertility is a threat to women's quality of life, it largely depends on their ability in responding to problems. Self-control is a psychological skill by which people can control their infertility-related emotions and come to a better understanding of their existential abilities that, ultimately, leads to personal peace and comfort [63]. In the studies conducted by Pasha et al. [64] Arsalan et al. [65], self-control reduced anxiety, depression, and stress in infertile women and improved the outcomes of treatment. By contrast, those who do not have enough knowledge about themselves and their abilities are at risk and, because of their weaknesses, do not have enough ability to do things, which can lead to their social isolation and reduces their quality of life [66].

Concerning the dimension of factors preventing adaptation, the mean score of the women was 60.98 ± 8.24 percent which was about 5 points lower than the mean total score of quality of life. At the Cairo International Conference on Population and Development in 1994, the problem of infertility was emphasized as an important health priority. However, this problem has been overlooked not only in developing countries but at most levels of international health management [67]. This view is a kind of underestimation of the infertility problem, which justifies the lack of governmental centers, lack of funding, specialists, and cost-effective treatment options [68]. Current disorganized infertility policies in the field of treatment and distribution have led to an inadequate distribution of public and private centers [69]. Many policies in Iran are only to increase the fertility rate, and economic problems in the provision of medicine and infertility treatment have unfortunately been overlooked. Current policies in Iran do not consider prevention, early diagnosis and referral, primary supportive care, and access to infertility services. These problems reduce the ability in coping with the problem of infertility [70].

Furthermore, because of patriarchal beliefs for the survival and continuity of the generation, women's reproductive inability can lead to gender-related inequalities. In such societies, infertility is considered a purely feminine issue, and, thus, women consider themselves inferior to men and are doubly pressured [11]. The salient difference between developed and developing societies in the issue of childbearing is that not having children in developed societies can be voluntary and considered an option for women to grow [71], but in developing countries, female infertility means a kind of disease and a feminine defective identity [72]. If men play their roles as partners and supporters of women, reproductive health indicators and treatment outcomes will surely be improved. As such, programs that involve men alongside women will be more successful [73].

It was revealed in our study that women with primary infertility have a lower quality of life. Similar results were obtained in the studies of Khayata et al. [74] and Shahraki et al. [75]. Because of having no child and experiencing no pregnancy, primary infertile women are more concerned than secondary infertile women, which can further affect the quality of life of these women with primary infertility [75].

As revealed in this study, the age of women, the age of the spouse, the duration of the marriage, and the duration of infertility are inversely correlated with the quality of life of infertile women. Women generally have better physical, mental, and environmental quality in the first 10 years of their marital life. However, with the passage of time and prolonged infertility, they grow a negative evaluation of themselves that may reduce their marital satisfaction and, consequently, have some negative effects on their quality of life [58]. Aging in men and women has a negative effect on the quality of life because it is a risk factor for reduced fertility and the chances of successful treatment [34, 37]. Similar findings have been found in different studies [76, 77], but it is noteworthy that education improves the quality of life of infertile women. Women's education is one of the most important factors which can empower women and make them use adaptive approaches in dealing with problems [78, 79]. Husbands' education makes them more aware of infertility, their wives feel less worried and their quality of life is higher [32]. Because most housewives are concerned about financing infertility treatment, improving their economic situation will reduce their worries about infertility treatment and can improve their quality of life [5, 22].

### Limitations of the study

The cross-sectional design of the present research was a limitation of the study as the direction of causality of the relationship may be questionable in this study. Given the fact that this study used for the first time the questionnaire of quality of life of infertile women with female factor, it was almost impossible to compare it accurately with other studies which had used different tools. Thus, as this tool is a valid and reliable one, it can be used in other studies in different societies.

## Conclusion

Assessment of the different aspects of the quality of life of infertile women including psychological effects, sexual life with infertility, family and social effects, infertility-related concerns, physical effects, adaptive approaches, and factors preventing infertility adaptation showed the lowest scores in the infertility-related concerns, and the sexual life aspects. Therefore, these are the most important dimensions of infertile women’s quality of life that need special consideration for intervention. Increased duration of marriage and duration of fertility decreases women's quality of life. Infertile women's quality of life is an important and challenging issue whose accurate measurement is critically significant. Given the use of a standard tool for assessing the quality of life of infertile women, the information extracted from this study can be used in planning and intervention for improving the quality of life of infertile women.

## Supplementary Information

**Additional file 1.**

## Data Availability

The data that support the findings of this study are available from Masoumeh Simbar but restrictions apply to the availability of these data, which were used under license for the current study, and so are not publicly available.

## Abbreviations

WHO: World Health Organization
SF-36: 36-Item Short Form Survey
HADS: Hospital Anxiety and Depression Scale
FertiQoL: Fertility Quality of Life Questionnaire
QOLICQ: Quality of Life in Infertile Couples Questionnaire
WHOQOL: World Health Organization Quality of Life
QOL-QIW: The quality of life questionnaire for infertile women
S-CVI: Scale- Content Validity Index
I-CVI: Item- Content Validity Index
VIF: Variance Inflation Index
SPSS: Statistical Package for the Social Sciences
SD: Standard Deviation

## References

[1] Olooto WE, Amballi AA, Banjo TA (2012). A review of Female Infertility; important etiological factors and management. J Microbiol Biotech Res.

[2] Hanson B, Johnstone E, Dorais J, Silver B, Peterson CM, Hotaling J (2017). Female infertility, infertility-associated diagnoses, and comorbidities: a review. J Assist Reprod Genet.

[3] Ombelet W, Cooke I, Dyer S, Serour G, Devroey P (2008). Infertility and the provision of infertility medical services in developing countries. Hum Reprod Update.

[4] Akhondi MM, Ranjbar F, Shirzad M, Ardakani ZB, Kamali K, Mohammad K (2019). Practical difficulties in estimating the prevalence of primary infertility in Iran. Int J Fertil Steril.

[5] Kiani Z, Simbar M, Hajian S, Zayeri F (2021). Quality of life among infertile women living in a paradox of concerns and dealing strategies: a qualitative study. Nurs Open.

[6] Bokaie M, Simbar M, Yassini Ardekani SM, Alavi-Majad H (2020). Does infertility influence couples’ relationships? A qualitative study. J Qual Res Health Sci.

[7] Matsubayashi H, Hosaka T, Izumi SI, Suzuki T, Makino T (2001). Emotional distress of infertile women in Japan. Human reproduction..

[8] Bokaie M, Simbar M, Ardekani SMY (2015). Sexual behavior of infertile women: a qualitative study. Iranian J Reprod Med.

[9] Onat G, Beji NK (2012). Effects of infertility on gender differences in marital relationship and quality of life: a case-control study of Turkish couples. Eur J Obstet Gyneco Reprod Biol.

[10] Drosdzol A, Skrzypulec V (2009). Evaluation of marital and sexual interactions of Polish infertile couples. J Sex Med.

[11] Hasanpoor-Azghdy SB, Simbar M, Vedadhir A (2015). The social consequences of infertility among Iranian women: a qualitative study. Int J Fertil Steril.

[12] Vitale SG, La Rosa VL, Rapisarda AMC, Lagana AS (2017). Psychology of infertility and assisted reproductive treatment: the Italian situation. J Psychosom Obstet Gynecol.

[13] Ashraf DM, Ali D, Azadeh DM (2014). Effect of infertility on the quality of life, a cross-sectional study. J Clin Diagn Res.

[14] World Health Organization (1998). The World Health Organization quality of life assessment (WHOQOL): development and general psychometric properties. Soc Sci Med.

[15] Chachamovich JL, Chachamovich E, Ezer H, Cordova FP, Fleck MM, Knauth DR (2010). Psychological distress as predictor of quality of life in men experiencing infertility: a cross-sectional survey. Reprod Health.

[16] Pessoa de Farias Rodrigues M, Lima Vilarino F, de Souza Barbeiro Munhoz A, da Silva Paiva L, de Alcantara Sousa LV, Zaia V (2020). Clinical aspects and the quality of life among women with endometriosis and infertility: a cross-sectional study. BMC women's health.

[17] Massarotti C, Gentile G, Ferreccio C, Scaruffi P, Remorgida V, Anserini P (2019). Impact of infertility and infertility treatments on quality of life and levels of anxiety and depression in women undergoing in vitro fertilization. Gynecol Endocrinol.

[18] Jahromi BN, Mansouri M, Forouhari S, Poordast T, Salehi A (2018). Quality of life and its influencing factors of couples referred to an infertility center in Shiraz. Iran Int J Fertil Steril.

[19] Luk BHK, Loke AY (2015). The impact of infertility on the psychological well-being, marital relationships, sexual relationships, and quality of life of couples: A systematic review. J Sex Marital Ther.

[20] Ware JE, Sherbourne CD (1992). The MOS 36-item short-form health survey (SF-36): I Conceptual framework and item selection. Med Care.

[21] Yaghmaei F, Mohammadi S, Alavi MH (2013). Developing and measuring psychometric properties of “quality of life questionnaire in infertile couples”. Int J Community Based Nurs Midwifery.

[22] Boivin J, Takefman J, Braverman A (2011). The fertility quality of life (FertiQoL) tool: development and general psychometric properties. Hum Reprod.

[23] Kiani Z, Simbar M, Hajian S, Zayeri F (2020). Development and psychometric evaluation of a quality of life questionnaire for infertile women: a mixed method study. Reprod Health.

[24] Goker A, Yanikkerem E, Birge O, Kuscu NK (2018). Quality of life in Turkish infertile couples and related factors. Hum Fertil.

[25] Namdar A, Naghizadeh MM, Zamani M, Yaghmaei F, Sameni MH (2017). Quality of life and general health of infertile women. Health Qual Life Outcomes.

[26] Madero S, Gameiro S, García D, Cirera D, Vassena R, Rodríguez A (2017). Quality of life, anxiety and depression of German, Italian and French couples undergoing cross-border oocyte donation in Spain. Hum Reprod.

[27] Fadaei M, Damghanian M, Rahimi-Kian F, Tehrani ESN, Mehran A (2016). The effect of educating based on continuous care model on the infertility treatment related quality of life. Nurs Pract Today.

[28] Lakatos E, Szigeti JF, Ujma PP, Sexty R, Balog P (2017). Anxiety and depression among infertile women: a cross-sectional survey from Hungary. BMC Womens Health.

[29] Direkvand-Moghadam A, Sayehmiri K, Delpisheh A, Kaikhavandi S (2014). Epidemiology of premenstrual syndrome (PMS)-a systematic review and meta-analysis study. J Clin diagn Res.

[30] Bornstein M, Gipson JD, Failing G, Banda V, Norris A (2020). Individual and community-level impact of infertility-related stigma in Malawi. Soc Sci Med.

[31] Peterson BD, Newton CR, Rosen KH, Skaggs G (2006). Gender differences in how men and women who are referred for IVF cope with infertility stress. Hum Reprod.

[32] Kiani Z, Simbar M, Hajian S, Hajian S, Zayeri F, Shahidi M, Saei Ghare Naz M (2020). The prevalence of anxiety symptoms in infertile women: a systematic review and meta-analysis. Fertil Res Pract.

[33] Kiani Z, Simbar M, Hajian S, Zayeri F (2021). The prevalence of depression symptoms among infertile women: a systematic review and meta-analysis. Fertil Res Pract.

[34] Lara LA, Fuentealba-Torres M, dos Reis RM, Cartagena-Ramos D (2018). Impact of Infertility on the Sexuality of Couples: an Overview. Curr Sex Health Rep.

[35] Tao P, Coates R, Maycock B (2011). The impact of infertility on sexuality: A literature review. Australas Med J.

[36] Purcell-Lévesque C, Brassard A, Carranza-Mamane B, Péloquin K (2019). Attachment and sexual functioning in women and men seeking fertility treatment. J Psychosom Obstet Gynecol.

[37] Tao P, Coates R, Maycock B (2012). Investigating marital relationship in infertility: a systematic review of quantitative studies. J Reprod Infertility.

[38] Cocchiaro T, Meneghini C, Dal Lago A, Fabiani C, Amodei M, Miriello D (2020). Assessment of sexual and emotional distress in infertile couple: validation of a new specific psychometric tool. J Endocrinol Invest.

[39] Tayebi N, Ardakani SMY (2007). The prevalence of sexual dysfunctions in infertile women. Middle East Fertil Soc J.

[40] Berger MH, Messore M, Pastuszak AW, Ramasamy R (2016). Association between infertility and sexual dysfunction in men and women. Sex Med Rev.

[41] Kohan S, Ghasemi Z, Beigi M (2015). Exploring infertile women's experiences about sexual life: A qualitative study. Iran J Nurs Midwifery Res.

[42] Piva I, Lo Monte G, Graziano A, Marci R (2014). A literature review on the relationship between infertility and sexual dysfunction: does fun end with baby making?. Eur J Contracept Reprod Health Care.

[43] Dyer SJ, Abrahams N, Hoffman M, van der Spuy ZM (2002). Men leave me as I cannot have children': women's experiences with involuntary childlessness. Hum Reprod.

[44] Alamin S, Allahyari T, Ghorbani B, Sadeghitabar A, Karami MT (2020). Failure in identity building as the main challenge of infertility: a qualitative study. J Reprod Infertility.

[45] Htay MNN, Donnelly M, Schliemann D, Loh SY, Dahlui M, Tamin NSBI (2020). Translation and Validation of the Breast Cancer Awareness Measurement Tool in Malaysia (B-CAM-M). Asian Pac J Cancer Prev.

[46] Rooney KL, Domar AD (2018). The relationship between stress and infertility. Dialogues Clin Neurosci.

[47] Yao H, Chan CHY, Chan CLW (2018). Childbearing importance: A qualitative study of women with infertility in China. Res Nurs Health.

[48] Ramezanzadeh F, Aghssa MM, Abedinia N, Zayeri F, Khanafshar N, Shariat M (2004). A survey of relationship between anxiety, depression and duration of infertility. BMC Womens Health.

[49] Hashemi S, Simbar M, Ramezani-Tehrani F, Shams J, Majd HA (2012). Anxiety and success of in vitro fertilization. Eur J Obstet Gynecol Reprod Biol.

[50] Boz I, Okumus H (2017). The, “everything about the existence” experiences of Turkish women with infertility: solicited diaries in qualitative research. J Nurs Res..

[51] Gerrits T, Van Rooij F, Esho T, Ndegwa W, Goossens J, Bilajbegovic A (2017). Infertility in the Global South: Raising awareness and generating insights for policy and practice. Facts Views Vis ObGyn.

[52] Rahimi MS, Fahami F, Najimi A (2017). The effectiveness of training through mobile on the practice of midwives in the management of pre-eclampsia. Biomed Pharmacol J.

[53] Maroufizadeh S, Ghaheri A, Almasi-Hashiani A, Mohammadi M, Navid B, Ezabadi Z (2018). The prevalence of anxiety and depression among people with infertility referring to Royan Institute in Tehran, Iran: a cross-sectional questionnaire study. Middle East Fertil Soc J.

[54] Keramat A, Masoomi SZ, Mousavi SA, Poorolajal J, Shobeiri F, Hazavhei SMM (2013). Quality of life and its related factors in infertile couples. J Res health Sci.

[55] Anokye R, Acheampong E, Mprah WK, Ope JO, Barivure TN (2017). Psychosocial effects of infertility among couples attending St. Michael’s Hospital, Jachie-Pramso in the Ashanti Region of Ghana. BMC Res Notes.

[56] Miller D (2013). Farmacology: HarperCollins.

[57] Sabarre K-A, Khan Z, Whitten AN, Remes O, Phillips KP (2013). A qualitative study of Ottawa university students’ awareness, knowledge and perceptions of infertility, infertility risk factors and assisted reproductive technologies (ART). Reprod Health.

[58] Bakhtiyar K, Beiranvand R, Ardalan A, Changaee F, Almasian M, Badrizadeh A (2019). An investigation of the effects of infertility on Women’s quality of life: a case-control study. BMC Womens Health.

[59] Jennings PK (2010). “God had something else in mind”: Family, religion, and infertility. J Contemp Ethnogr.

[60] Taghipour A, Karimi FZ, Latifnejad Roudsari R, Mazlom SR (2020). Coping strategies of women following the diagnosis of infertility in their spouses: A qualitative study. Evid Based Care.

[61] Martins MV, Peterson B, Almeida V, Mesquita-Guimarães J, Costa M (2014). Dyadic dynamics of perceived social support in couples facing infertility. Hum Reprod.

[62] Slade P, O'Neill C, Simpson AJ, Lashen H (2007). The relationship between perceived stigma, disclosure patterns, support and distress in new attendees at an infertility clinic. Hum Reprod.

[63] Cunha M, Galhardo A, Pinto-Gouveia J (2016). Experiential avoidance, self-compassion, self-judgment and coping styles in infertility. Sex Reprod Healthcare.

[64] Pasha H, Faramarzi M, Esmailzadeh S, Kheirkhah F, Salmalian H (2013). Comparison of pharmacological and nonpharmacological treatment strategies in promotion of infertility self-efficacy scale in infertile women: a randomized controlled trial. Iran J Reprod Med.

[65] Arslan-Özkan İ, Okumuş H, Buldukoğlu K (2014). A randomized controlled trial of the effects of nursing care based on Watson's Theory of Human Caring on distress, self-efficacy and adjustment in infertile women. J Adv Nurs.

[66] Cousineau TM, Domar AD (2007). Psychological impact of infertility. Best Pract Res Clin Obstet Gynaecol.

[67] Widge A, Cleland J (2009). The public sector's role in infertility management in India. Health Policy Plan.

[68] Nachtigall RD (2006). International disparities in access to infertility services. Fertil Steril.

[69] Zuccala E, Horton R (2018). Addressing the unfinished agenda on sexual and reproductive health and rights in the SDG era. Lancet.

[70] Morshed-Behbahani B, Lamyian M, Joulaei H, Montazeri A (2020). Analysis and exploration of infertility policies in Iran: a study protocol. Health Res Policy Syst.

[71] Greil AL, McQuillan J, Johnson K, Slauson-Blevins K, Shreffler KM (2010). The hidden infertile: infertile women without pregnancy intent in the United States. Fertil Steril.

[72] Kiani Z, Simbar M (2019). Infertility’s Hidden and Evident Dimensions: A Concern Requiring Special Attention in Iranian Society. Iran J Public Health.

[73] Kura S, Vince J, Crouch-Chivers P (2013). Male involvement in sexual and reproductive health in the Mendi district, Southern Highlands province of Papua New Guinea: a descriptive study. Reprod Health.

[74] Khayata G, Rizk D, Hasan M, Ghazal-Aswad S, Asaad M (2003). Factors influencing the quality of life of infertile women in United Arab Emirates. Int J Gynecol Obstet.

[75] Shahraki Z, Ghajarzadeh M, Ganjali M (2019). Depression, anxiety, quality of life and sexual dysfunction in Zabol women with infertility. Mædica.

[76] Zurlo MC, Della Volta MFC, Vallone F (2018). Predictors of quality of life and psychological health in infertile couples: the moderating role of duration of infertility. Qual Life Res.

[77] Kiani Z, Simbar M, Dolatian M, Zayeri F (2018). Women’s empowerment in reproductive decision-making needs attention among Iranian women. Iran J Public Health..

[78] Kiani Z, Simbar M, Dolatian M, Zayeri F (2020). Structural equation modeling of psychosocial determinants of health for the empowerment of Iranian women in reproductive decision making. BMC Womens Health.

[79] Alishah A, Ganji J, Mohammadpour R, Kiani Z, Shahhosseini Z (2019). Women’s Reproductive Empowerment: A Comparative Study of Urban and Rural Females in Iran. Int J Women’s Health Reprod Sci.

