# Supplementary material for: Investigating different dimensions of infertile women’s quality of life: a descriptive cross-sectional study
Source: BMC Public Health. 2022 Dec 27;22:2436. doi: 10.1186/s12889-022-14924-w (PMC9793385; doi:10.1186/s12889-022-14924-w)
Supplement: Supplementary file 1 — Additional file 1. [file 12889_2022_14924_MOESM1_ESM.docx]

| No | Items | Never | Rarely | Sometimes | Often | Always |
| --- | --- | --- | --- | --- | --- | --- |
| 1 | I feel lonely after infertility |  |  |  |  |  |
| 2 | Not being a mother, I think there is a lack in my life |  |  |  |  |  |
| 3 | I feel sad watching the lives of those around me |  |  |  |  |  |
| 4 | After infertility, I am irritable |  |  |  |  |  |
| 5 | My sexual desire is lower than before |  |  |  |  |  |
| 6 | The number of times I have sex is lower than before |  |  |  |  |  |
| 7 | The timing of sex has disrupted our sexual relation because of the infertility treatment process |  |  |  |  |  |
| 8 | I have less emotional relationship with my husband |  |  |  |  |  |
| 9 | I hide my infertility because of the misjudgment of others |  |  |  |  |  |
| 10 | The relationship with my friends is less than before |  |  |  |  |  |
| 11 | The high costs of infertility treatment worry me |  |  |  |  |  |
| 12 | I am worried about the side effects the drugs |  |  |  |  |  |
| 13 | I am worried that assisted reproductive techniques will harm the fetus in future |  |  |  |  |  |
| 14 | I am worried about the effect of aging on my fertility |  |  |  |  |  |
| 15 | I am worried about the recurrence of treatment |  |  |  |  |  |
| 16 | I am weak and lethargic as I am the cause of infertility |  |  |  |  |  |
| 17 | After taking infertility drugs, I suffer from digestive problems (bloating, nausea, diarrhea, etc.) |  |  |  |  |  |
| 18 | I have neurological symptoms (headache and dizziness) after taking infertility drugs |  |  |  |  |  |
| 19 | The support of the treatment team calms me down |  |  |  |  |  |
| 20 | Talking to other infertile people calms me down |  |  |  |  |  |
| 21 | I feel better by getting rid of negative thoughts |  |  |  |  |  |
| 22 | Perceiving the value of life and being together makes me calm |  |  |  |  |  |
| 23 | The government's lack of financial support for infertility has made adaptation to infertility difficult for me |  |  |  |  |  |
| 24 | My husband's lack of awareness of infertility upsets me |  |  |  |  |  |
| 25 | My family's lack of awareness of infertility upsets me |  |  |  |  |  |

**The Quality of life questionnaire for infertile women -25 (QOL-QIW -25)**

Quality of life questionnaire for infertile women (QOL-QIW -25) was designed to measure infertile women's quality of life. This questionnaire consists of 7 factors as follows: 1) Psychological effects with 4 items; 2) Sexual life with infertility with 3 items; 3) Family and social effects with 3 items; 4) Infertility-related concerns with 5 items; 5) Physical effects with 3 items; 6) Adaptive approaches with 4 items; and 7) Factors preventing adaptation with 3 items.

QOL-QIW-25 consists of 25 terms which are measured using a 5-point scale including never (5), rarely (4), sometimes (3), often (2) and always (1). The total score for the factors and the whole instrument is calculated and standardized using the following formula and based on 0 to 100:

$$Score in percent= \frac{The raw score obtained - Minimum possible score}{Maximum possible score - Minimum possible score}\times100$$

Questions 19, 20, 21 and 22 are scored reversely.

QOL-QIW-25 was developed with the means of 0.94 and 0.92 for S-CVI and I-CVI, respectively. Reliability of the questionnaire was confirmed with the Cronbach's alpha of 0.87 and intra-cluster correlation of 0.97. This questionnaire is used to assess the quality of life of infertile women in general and its challenges based on the 7 mentioned factors. It can be used in research and counseling as well as care services to promote the health and quality of life of infertile women
